# Accurate Expression Profiling of Very Small Cell Populations

**DOI:** 10.1371/journal.pone.0014418

**Published:** 2010-12-28

**Authors:** Eva Gonzalez-Roca, Xabier Garcia-Albéniz, Silvia Rodriguez-Mulero, Roger R. Gomis, Karl Kornacker, Herbert Auer

**Affiliations:** 1 Institute for Research in Biomedicine, Barcelona, Spain; 2 Medical Oncology, Hospital Clínic de Barcelona, Barcelona, Spain; 3 Institut Català de Recerca i Estudis Avançats, Barcelona, Spain; 4 Division of Sensory Biophysics, The Ohio State University, Columbus, Ohio, United States of America; University of Calgary, Canada

## Abstract

**Background:**

Expression profiling, the measurement of all transcripts of a cell or tissue type, is currently the most comprehensive method to describe their physiological states. Given that accurate profiling methods currently available require RNA amounts found in thousands to millions of cells, many fields of biology working with specialized cell types cannot use these techniques because available cell numbers are limited. Currently available alternative methods for expression profiling from nanograms of RNA or from very small cell populations lack a broad validation of results to provide accurate information about the measured transcripts.

**Methods and Findings:**

We provide evidence that currently available methods for expression profiling of very small cell populations are prone to technical noise and therefore cannot be used efficiently as discovery tools. Furthermore, we present Pico Profiling, a new expression profiling method from as few as ten cells, and we show that this approach is as informative as standard techniques from thousands to millions of cells. The central component of Pico Profiling is Whole Transcriptome Amplification (WTA), which generates expression profiles that are highly comparable to those produced by others, at different times, by standard protocols or by Real-time PCR. We provide a complete workflow from RNA isolation to analysis of expression profiles.

**Conclusions:**

Pico Profiling, as presented here, allows generating an accurate expression profile from cell populations as small as ten cells.

## Introduction

Microarray technology provided the first opportunity to simultaneously study the expression of thousands of genes [1]. Developments over recent years have allowed researchers to extend the interrogation of expression to all known genes of a certain organism using a single microarray. Today, massive parallel sequencing allows transcriptomic analysis without the necessity of previously identified transcripts [2]. Standard methods for expression profiling use micrograms of total RNA [3], the equivalent of millions of cells [4]. Given the large numbers of cells required for expression profiling purposes, standard methods have limited use in many areas of biology. A mouse, for example, has 5,000 hematopoietic stem cells, of which approximately 100 actively divide [5]. A comprehensive evaluation of the physiological state of these 100 cells by expression profiling is currently not possible with standard methods. Other reasons might impede the use of high numbers of cells; a recent study of human pre-implantation development used almost 200 human embryos to provide information about expression profiles of just four developmental stages [6]. Due to ethical issues, a study of this nature is unlikely to be reproduced in many countries.

To obtain sufficient signal on microarrays or to obtain sufficient material for massive parallel sequencing from limited cell numbers, cDNA amplification methods have been developed, which were intended to represent the relative abundance of different transcripts in their amplification products. Twenty years ago, expression analysis of several genes was proposed for the first time to be doable from an individual cell [7]. The in vitro transcription (IVT) based method described there can be used in two cycles of cDNA synthesis and IVT and it is currently broadly used for expression profiling from nanograms of RNA [8], the equivalent of 1,000 or more cells. Commercial providers of amplification chemistries based on two rounds of IVT also recommend to start from at least nanograms of RNA [9].

Another method to generate large amounts of cDNA from nanograms of RNA works based on logarithmic amplification (Transplex, http://www.rubicongenomics.com/products/transplex). Transplex performs fragmentation before amplification to overcome differences in amplification efficiencies due to different lengths of transcripts. An independent evaluation [10] of Transplex observed good comparability of differential expression to non-amplified RNA when Transplex used RNA equivalents of 1,000 or more cells (12 to 300 ng total RNA). As mentioned by the authors, “hybridizations from <10 ng of input total RNA had decreased correlation”. The extend of decrease was not described [10].

Several years ago, an isothermal, linear nucleic acid amplification method became available [11], which is now widely used for RNA amounts in the range of nanograms [8]. The amplified cDNA generated by this single primer isothermal amplification procedure (SPIA) provides less crosshybridization on microarrays than the frequently used cRNA [12] and therefore provides higher specificity. An independent evaluation [13] of SPIA amplification from picograms of RNA observed thousands of probe sets to measure the opposite direction of differential expression compared to the standard protocol recommended by the microarray manufacturer.

Another logarithmic amplification approach (SMART PCR) introduces adapter sequences to both ends of the cDNA during synthesis for subsequent amplification [14]. The original commercial version of SMART chemistry is suggested to be used with 100 ng RNA as starting material (http://www.clontech.com/images/pt/PT3751-1.pdf). An independent evaluation [15] of SMART amplification from picograms of RNA observed high false positive and false negative rates compared to the standard protocol recommended by the microarray manufacturer.

Almost ten years ago a PCR based global cDNA amplification method was developed, which uses a single oligo(dT) containing primer for exponential amplification [16]. This approach claims to allow the quantification of global gene expression of very few or even individual cells. It was later optimized several times [17], [18], [19], [20]. This PCR based amplification is currently the method of choice for expression profiling of very small cell populations [21], [22], [23]. Here we report that latest optimizations of this method [17], [18], [20] do not dramatically reduce false positive or false negative measurements. High rates of false positives reduce the power of expression profiling since only very dramatic alterations in expression can be reliably detected. High rates of false negatives impede interrogation of the complete transcriptional status of a cell type.

To be useful as a discovery tool, an expression profiling method for characterization of very small cell populations should fulfill the following criteria: 1) Dilutions of RNAs should provide results of differential expression comparable to standard protocols using much higher amounts of RNA across the entire transcriptome. 2) The measurements from diluted RNA should correlate comparably well to Real-time PCR (qPCR) as the expression profiles from standard protocols do. This criterion should be evaluated for a broad range of transcripts. 3) Variability of technical replicates (repeated processing of aliquots of the same RNA preparation) must be small so that differential expression measured between samples is most likely to represent biological differences between samples. 4) Once these criteria are fulfilled using dilutions of RNAs purified by standard protocols, RNAs purified from small cell populations should be analyzed and compared to results from bigger populations. The average measurements of several small populations should provide results similar to those of a big population.

We describe a novel workflow for expression profiling which we call Pico Profiling. It contains RNA isolation from very small cell populations, cDNA synthesis and amplification, labeling of cDNA using biotin and hybridization to expression arrays from Affymetrix. Pico Profiling uses Whole Transcriptome Amplification (WTA, http://www.sigmaaldrich.com/etc/medialib/docs/Sigma/Bulletin/wta1bul.Par.0001.File.tmp/wta1bul.pdf) to generate sufficient cDNA for microarray expression analysis. WTA is a chemistry based on Transplex (described above), with improved coverage for transcriptome amplification. It obtains similar, if not identical, information about expression from a few cells to that gained from millions of cells by standard protocols. Our results were validated by comparison with standard expression profiling performed in our own laboratory and elsewhere. In addition, we show that our measurements are consistent with qPCR measurements, the gold standard of transcript quantification across over 800 genes. A complete workflow is presented from RNA isolation from individual cells through amplification to microarray analysis to interrogate the entire transcriptome from as few as ten cells. Formerly available methods for expression profiling usually had to apply filtering criteria like signal intensity, “Present” calls and stringent cutoffs for high levels of differential expression to avoid false-positive calls. Consequently, these methods could not provide a comprehensive overview across all measured transcripts. We show that the rates of outliers and abnormal measurements of Pico Profiling are so low that no filtering criteria against false-positive measurements have to be applied. Therefore, interrogation of the entire transcriptome can be performed from very small cell populations.

## Results

### Establishment and evaluation of WTA for genome-wide expression profiling

**Figure 1:**
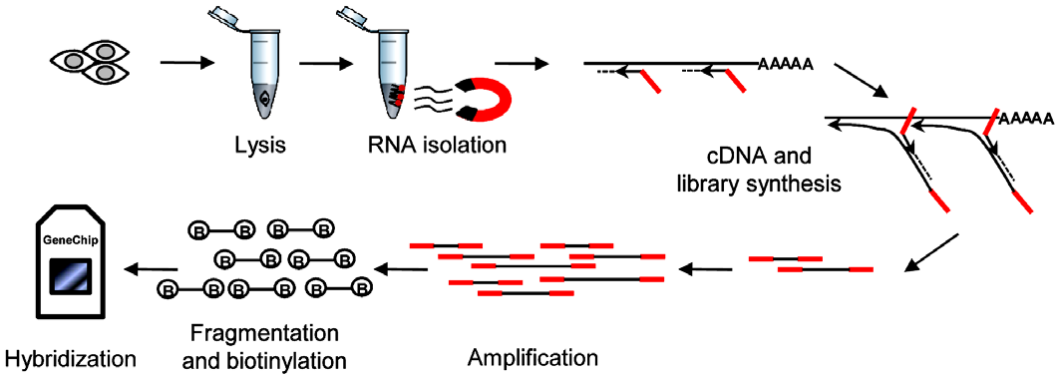
Workflow of Pico Profiling. Cells are lysed, RNA is purified by magnetic beads, cDNA is synthesized followed by library preparation and amplification; after column purification, cDNA is fragmented and biotinylated, followed by hybridization to a microarray.

WTA was originally described for expression profiling of nanograms of total RNA (http://www.sigmaaldrich.com/etc/medialib/docs/Sigma/Bulletin/wta1bul.Par.0001.File.tmp/wta1bul.pdf). WTA uses cDNA synthesis library preparation, adaptor ligation and PCR amplification to generate micrograms of cDNA (Figure 1). To our knowledge, only limited information is available about false positive/negative measurements after Transplex amplification relative to standard procedures for expression profiling [10], [24]; moreover, broad validation by qPCR has not been performed. For WTA, a modified version of Transplex amplification, no validation is available at all. As a first evaluation of WTA, nanogram amounts of RNA were used for amplification, as recommended by the distributor (Sigma Aldrich). We fragmented the amplified cDNA to generate higher numbers of DNA ends, which were subsequently labeled by biotin. The amplified and labeled cDNA was hybridized to Affymetrix Human Gene ST 1.0 arrays. A detailed description of the sample processing protocol is available in File S1.

We evaluated WTA with the samples A and B (Universal Reference RNA, Stratagene and Human Brain RNA, Ambion respectively) of the Microarray Quality Control (MAQC) study [3], which have been analyzed on over 1,500 microarrays and for which almost 1,000 transcripts have been measured by alternative methods like qPCR. Results of the WTA-based expression profiles were first compared with those obtained for identical samples on the same microarrays in a different laboratory, which used the sample processing procedure recommended by the array manufacturer (Affymetrix) [25]. We also compared our profiles with results from qPCR and microarrays included in the MAQC study.

**Figure 2:**
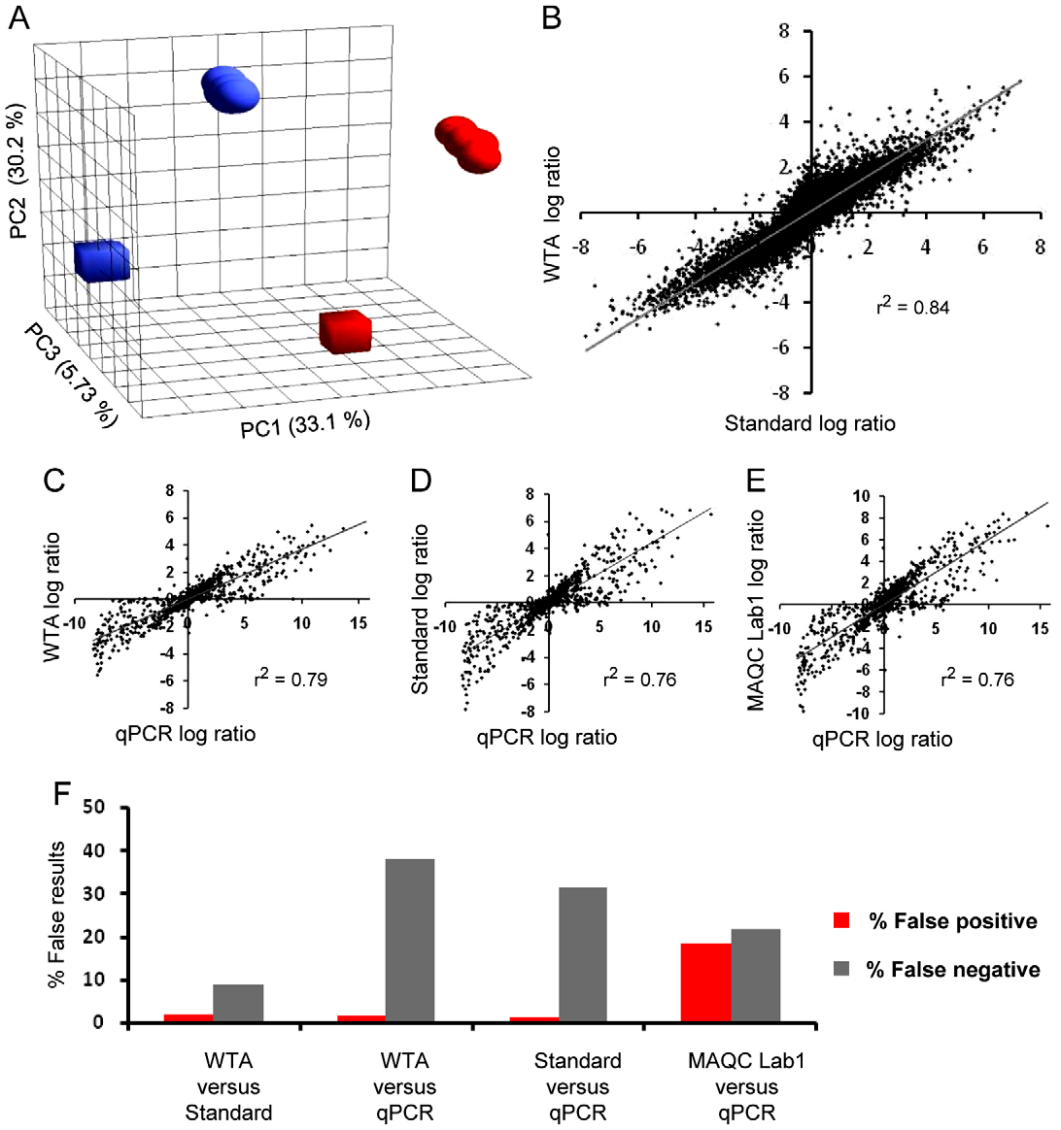
Evaluation of WTA for expression profiling. *A*, PCA of expression profiles of MAQC samples A (red) and B (blue) processed in triplicate following the manufacturer's recommendations (cubes) and processed seven times on different days using WTA (globes); the contribution of the specific component is shown next to its axis. *B*, correlation of differential expression between MAQC samples A and B measured by the manufacturer's method and using WTA; average values of triplicates are displayed and all measurements of all probe sets are displayed. *C to E*, correlation of differential expression measured by (*C*) qPCR versus WTA, (*D*) qPCR versus the standard method and (*E*) qPCR versus laboratory 1 of the original MAQC study. *F*, Quantification of false positive and false negative rates for several expression profiling methods; from left to right: for WTA versus the manufacturer's protocol, for WTA versus qPCR, for the manufacturer's protocol versus qPCR, and for microarray measurements from the original MAQC study versus qPCR.

Principle Component Analysis (PCA) of WTA-based expression profiles from nanograms of RNA, analyzed on different days and from slightly different amounts of RNA, separated the samples in a similar direction and distance as when processed following the microarray manufacturer's recommendations (Figure 2A). Results of differential expression across all probe sets on the Gene ST array, measured by the standard protocol and by WTA-based expression profiling, correlated well with each other (Figure 2B). Next we compared our differential expression profiles with measurements by qPCR. For this purpose, qPCR data from the MAQC study were used. Our differential expression profiles correlated slightly better with qPCR than those generated following the recommendations of the microarray manufacturer and slightly better than microarray data generated in the MAQC study, in which a former type of Affymetrix expression arrays (U133Plus2.0) was used (Figure 2C–E).

### A uniform analysis method to quantify false positive and false negative measurements and outliers across data sets

To compare measurements across a range of amplification methods, several microarray platforms and other quantification methods, such as qPCR and massive parallel sequencing, we used a uniform method to analyze performance. Thus we were able to directly compare the results of the methods with each other. We defined one method as the reference method (for example expression profiles from micrograms of RNA, processed according to standard protocols) and another measurement as the one under evaluation (expression profiles from picograms of RNA, processed according to alternative protocols). In other comparisons, qPCR is the reference method and microarrays data (or sequencing data) is under evaluation. We used triplicates of results for each of the methods (except for the data of Tang et al. [18], where sequencing was performed without replicates and qPCR was performed in duplicate). When more than three replicates were available for a certain method (for example Affymetrix U133Plus2.0 microarray data from the MAQC study), the first three replicates were used (for example replicates one to three from laboratory one of the MAQC study).

For each comparison, the reference method was first used to partition all measured transcripts into three groups, positive, negative and other. The measurements from the evaluated method were then used to partition the positives into true positives, false negatives and other. Similarly, the negatives were partitioned into true negatives, false positives and other. The partitioning criteria are described in Material and Methods.

Compared to the method based on the microarray manufacturer's recommendations, WTA-based expression profiling generated less than 2% false positive and 9% false negative measurements respectively (Figure 2F). Much larger discrepancies are known to occur when distinct amplification and labeling methods are used [8].

Compared to qPCR, WTA-based expression profiling generated 0.2% false positive measurements and 27% false negative measurements (Figure 2F), minimally more than the method recommended by the microarray manufacturer. The original microarray data from the MAQC study showed higher false positive rates and slightly lower false negative rates. In summary, we concluded that WTA-based expression profiling from nanograms of RNA generates measurements of differential expression robustly and comparable to the quality of other amplification and labeling methods.

### Pico Profiling from RNA equivalents of a few cells

The yield of total RNA of many types of cells propagated in cell culture is in the range of 10 pg [4]. Since our aim was to measure expression profiles from very few cells, we tested whether WTA-based expression profiling provides reliable results from 100 pg of total RNA and how well these results compare to measurements from RNA equivalents of much larger cell populations, namely nanograms to micrograms of total RNA.

**Figure 3:**
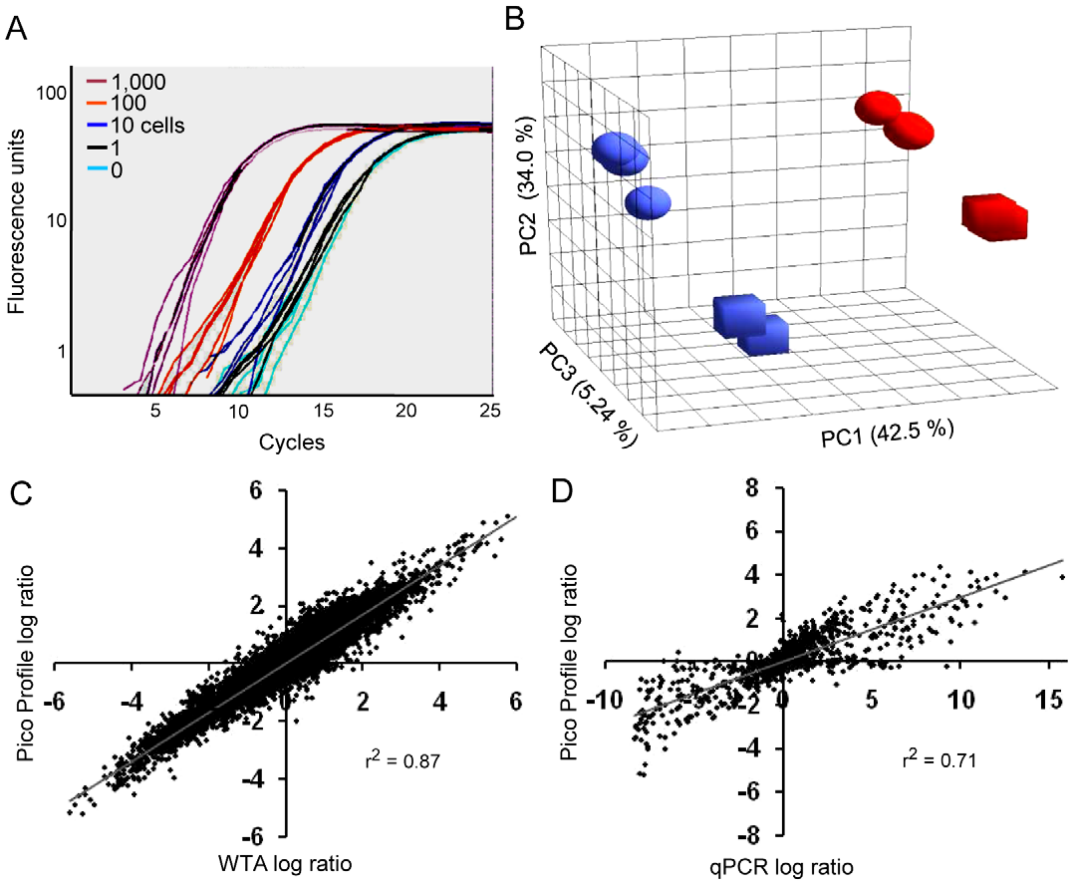
Evaluation of expression profiles from pg of RNA (Pico Profiling). *A*, SYBR Green amplification signals from 1,000, 100, 10, 1 and 0 cells. *B*, PCA of expression profiles of MAQC samples A (red) and B (blue) processed in triplicate starting from 25 ng RNA (cubes) and 100 pg (globes); the contribution of the specific component is shown next to its axis. *C*, correlation of differential expression between MAQC samples A and B measured from 25 ng and 100 pg RNA; average values of triplicates are displayed, all measurements of all probe sets are displayed. *D*, correlation of differential expression measured by Pico Profiling versus qPCR.

The WTA protocol of the distributor recommends 17 cycles of amplification for nanograms of RNA, but more cycles can be applied when lower amounts are used. To prevent amplification above the linear range, we added SYBR Green to the amplification reaction in order to monitor efficiency in real-time. The amplification reaction was interrupted once the SYBR Green signal reached a plateau (Figure 3A). At the beginning of this phase, sufficient cDNA had been generated for microarray hybridization purposes. This occurred for the entire range of starting amounts of RNA tested. WTA generates a certain amount amplified DNA even without input RNA, as many other amplification methods do [13]. When RNA amounts between zero and 1,000 pg were used and cDNA was quantified after identical numbers of amplification cycles, cDNA yield was dependent on the amount of starting material (Table S1). cDNA amplified without input RNA shows lower molecular weight than products generated from RNA (Figure S1). This amplification product could be caused by random extension of primer dimers and seems not to interfere with downstream applications. When cDNA was generated without input RNA and was hybridized to microarrays, signals merely raised above background (Figure S2).

For the MAQC samples, 100 pg total RNA was amplified for 23 cycles and the amplification product was again analyzed on Gene ST microarrays. In PCA, Pico Profiling of 100 pg RNA separated the samples in a similar direction and distance as profiles generated from 25 ng RNA (Figure 3B) with slightly higher variability between replicates. Measurements of transcripts that showed strong differences between replicates on average had only half the intensity of truly differentially expressed genes (data not shown). Results of differential expression across all the probe sets on the Gene ST array measured from 25 ng and 100 pg correlated well with each other (Figure 3C). Differential expression from Pico Profiling correlated only slightly lower with qPCR (Figure 3D) than differential expression from WTA-based expression profiles from nanograms RNA (Figure 2C). The reduced correlation affected high-copy-number transcripts to a lesser extent than it affected low-copy number transcripts (Figure S3).

### Benchmarking Pico Profiling relative to methods currently available for picograms of RNA

We compared the performance of Pico Profiling with three variations of the formerly published PCR based global cDNA amplification method [16] (chronologically listed by publication date). Kurimoto et al. [17] performed expression profiling on 10 pg RNA and compared the results with the standard protocol; Jensen and Watt [20] performed expression profiling from 50 pg RNA prepared from two cell lines and compared results with data from the standard protocol, as published earlier [26]; and Tang et al. [18] conducted qPCR and expression profiling by massive parallel sequencing on the RNA of one oocyte, representing approximately 250 pg RNA [27]. The results are referred to by the names of the first authors. In Kurimoto's data set, expression profiles of only one sample were compared between a range of amounts of starting material. Consequently, neither true differential expression between two samples nor false positive rates could be calculated. To compare our profiling results with Kurimoto's data set, a deviant comparative analysis strategy was performed. This strategy and the results are described in a later paragraph. To provide a comprehensive comparison to formerly available profiling methods for small amounts of RNA, we also compared Pico Profiling to data available in the literature about performance of SMART PCR [15], Ribo SPIA [13] and another variation of global cDNA amplification [15].

**Figure 4:**
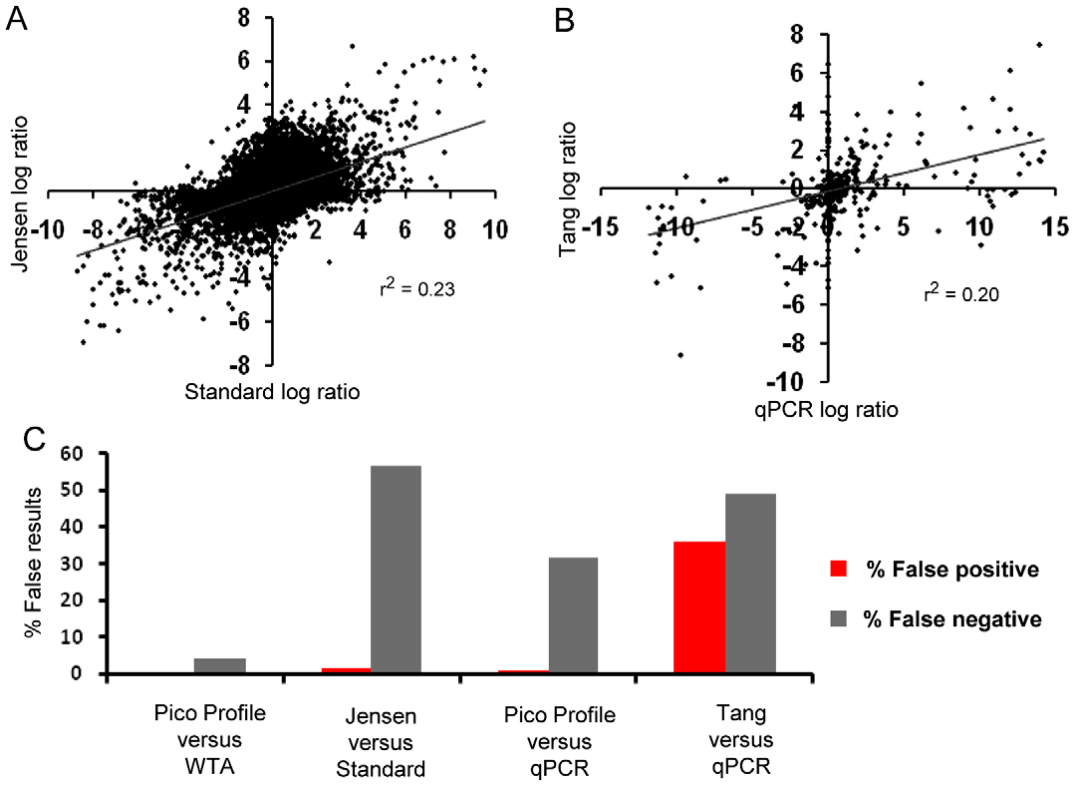
Comparison of Pico Profiling with formerly available profiling methods from small amounts of RNA. *A*, correlation of differential expression measured from 50 pg RNA versus 5 µg in Jensen's data set. *B*, correlation of differential expression measured by sequencing versus qPCR in Tang's data set. *C*, quantification of false positive and false negative rates for different expression profiling methods; from left to right: for Pico Profiling versus WTA from 25 ng, for 50 pg RNA versus 5 µg in Jensen's data, for Pico Profiling versus qPCR, and for sequencing versus qPCR in Tang's data set.

Correlation of differential expression measured by WTA-based expression profiling from nanograms and Pico Profiling from picograms of RNA (Figure 3C) was much higher than in Jensen's data set (Figure 4A) when comparing the standard method from micrograms RNA versus that measured from picograms RNA. The correlation of Pico Profiling versus qPCR was also much higher than correlation of sequencing results versus qPCR in Tang's data set (Figs. 3D and 4D respectively).

False positive and false negative rates of Pico Profiling from 100 pg RNA were less than 1 and 4% respectively, compared to measurements by WTA-based expression profiling from 25 ng (which we used as the reference method here) (Figure 4C). In Jensen's data set, measurements from picograms of RNA produced less than 2% false positive measurements but over 50% false negatives compared to the standard method.

When compared to measurements from qPCR, false positive and false negative rates of Pico Profiling were less than 2 and 39% respectively (Figure 4C). These results indicate a slight increase in false measurements when compared to qPCR than expression profiles from 25 ng (Figure 2F). In contrast, when the expression profiles generated by massive parallel sequencing from Tang's data set was compared with their qPCR results, 36% of measurements of differential expression were false positive and 49% of the genes measured as differentially expressed by qPCR were not detected by sequencing (Figure 4C).

**Figure 5:**
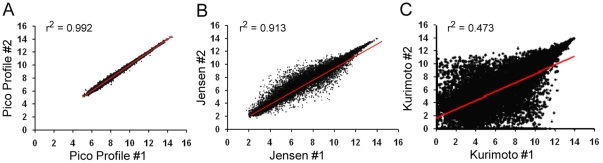
Correlation of expression estimates of expression profiling from picograms RNA. *A*, correlation of replicates of Pico Profiling from 100 pg RNA. *B*, correlation of replicates from Jensen's data set from 50 pg. *C*, correlation of replicates from Kurimoto's data set from 10 pg. Expression values are expressed in log2 scale and all measurements of all probe sets are displayed.

Next, we compared the reliability of our expression profiles with Kurimoto's data set. Since Kurimoto used only one RNA sample for comparison of expression profiles from picograms versus micrograms, we compared correlation of technical replicates. Pico Profiling showed much higher correlation of replicates (Figure 5A) than replicates of Kurimoto's data set (Figure 5C), and also outperformed Jensen's expression profiles for correlation between replicates (Figure 5B). The differences of lowest intensities, i.e. background intensities, observed in these three data sets is caused by the different normalization methods (quantile scaling (WPP) for Pico Profiling, quantile normalization (RMA) for Jensen's data and modeling (dChip) for Kurimoto's data). Exchanging the normalization methods between data sets changed the background intensities but changed the variability of technical replicates only to a small extend (data not shown).

To assess sensitivity and technical variability of different profiling methods from small amounts of RNA, we also analyzed frequencies of outliers. Outliers were defined as measurements of differential expression between individual replicates where pairs of samples showed different directions and at least one of them showed a magnitude greater than two (for details, see Material and Methods). Analysis of outlier rates showed that Pico Profiling provides minimal outliers (Tables 1 and 2). Consequently, we could perform analysis of correlation of differential expression between Pico Profiling and larger amounts of RNA from the average of triplicates without filtering against outliers.

**Table 1:** Comparison of expression profiling results generated by standard protocols versus small amounts of RNA.

Reference method	Method under evaluation	% True positive	% False positive	% True negative	% False negative	% Positive outliers	% Negative outliers	r^2^ of differential expression
Affymetrix chemistry (100 ng)^1^	WTA (25 ng)	91.2	1.8	98.2	8.8	0.0	0.0	0.84
WTA (25 ng)	Pico Profiling (100 pg)	94.3	0.3	98.3	3.9	1.8	1.4	0.87
WTA (2,000 cells)	Pico Profiling (10 cells)	97.5	0.1	98.5	1.25	1.25	1.4	0.75
Affymetrix chemistry (5,000 ng)^2^	Jensen (50 pg)^3^	25.3	1.9	90.0	56.6	18.1	8.1	0.23
Affymetrix chemistry (5,000 ng)^4^	Kurimoto (10 pg)^4^	N.A.	2.1	36.7	N.A.	N.A.	61.2	N.A.
Standard protocol (100 µg)^5^	Global amplification (10 pg)^5^	39.3 7	60.7 7	N.A.	77.5 7	N.A.	N.A.	0.36 8
Standard protocol (100 µg)^5^	SMART PCR (10 pg)^5^	70.7 7	29.3 7	N.A.	88.3 7	N.A.	N.A.	0.56 8
Affymetrix chemistry (2,000 ng)^6^	Ribo-SPIA (500pg)^6^	N.A.	N.A.	N.A.	N.A.	N.A.	N.A.	0.10 9

1 Data calculated from CEL files of [24];

2 Data calculated from CEL files of [26];

3 Data calculated from CEL files of [19];

4 Data calculated from dChip estimates of [16];

5 Data from [15];

6 Data from [13];

7 Data calculated from Table 4 of [15];

8 Data calculated from Figure 4 of [15];

9 Data calculated from Figure 6 of [13]; details of analysis methods are described in Material and Methods.

**Table 2:** Comparison of expression profiling results generated from small amounts of RNA versus qPCR.

Reference method	Method under evaluation	% True positive	% False positive	% True negative	% False negative	% Positive outliers	% Negative outliers	r^2^ of differential expression
qPCR^1^	Affymetrix chemistry (5,000 ng)^1^	78.1	18.5	81.5	21.7	0.0	0.0	0.76
qPCR^1^	Affymetrix chemistry (100 ng)^3^	68.5	1.1	98.9	31.5	0.0	0.0	0.76
qPCR^1^	WTA (25 ng)	61.7	1.4	98.6	38.1	0.2	0.0	0.79
qPCR^1^	Pico Profiling (100 pg)	60.4	1.5	97.6	38.7	0.9	1.0	0.71
qPCR^2^	SOLiD sequencing (200 pg)^2^	43.3	36.2	63.8	49.0	7.7	N.A.	0.20

1 Data calculated from qPCR results and CEL files of [3];

2 Data calculated from CEL files of [24];

3 Data calculated from Supplementary Table 3 of [18].

True positive and true negative rates of Pico Profiling versus larger amounts of starting material were always above 95% while formerly published methods only detected between 25 and 71% of truly differentially expressed genes (Table 1). False positive rates stayed below 0.3% for Pico Profiling while alternative methods generated between 2 and 71% false positive measurements. False negative rates of Pico Profiling always stayed below 5% while formerly published methods failed to detect between 57% and 88% of truly differentially expressed genes.

When microarray measurements of differential expression are compared to qPCR, it is well known that microarrays provide a smaller dynamic range [3]. This phenomenon causes lower rates of true positive measurements when microarray data is compared to qPCR (Table 2). Although Pico Profiling also suffered from this compression of dynamic range, it still provides far more true positive measurements than SOLiD sequencing of picograms of RNA (60% versus 43% respectively). Compared to qPCR, Pico Profiling generates less than 2% false positives while SOLiD sequencing provides 36%. Due to our knowledge, for the other published methods of profiling from small amounts of RNA, no large scale comparison to qPCR is available. For comparison, we also included in Table 1 data from other studies that evaluated profiling methods from small amounts of RNA.

### Expression profiling from 10 cells

To allow expression profiling from very few cells, we developed a strategy for RNA isolation from individual cells. Cells were sorted directly into lysis buffer, followed by RNA isolation by magnetic beads. We first used this RNA isolation method on thousands of cells to quantify the amount of RNA that can be purified per cell. Across several cell lines, we purified approximately 10 pg of total RNA per cell (Table S2) and the differences in RNA yield among the cell lines were reproducible (data not shown). RNA integrity analyzed by capillary electrophoresis was high (Figure S4) and indistinguishable from RNA integrity obtained by standard methods of isolation. The entire procedure from RNA isolation to the measurement of expression profiles is outlined in Figure 1.

Primary tumor and metastatic cell line derivatives (SW480 and SW620, ATCC# CCL-228 and CCL-227 respectively) from the same patient were used. Ten cells of each line in the G1 phase of the cell cycle were sorted and RNA was purified. After 23 cycles of WTA and hybridization to Gene ST arrays, expression profiles were compared with those generated from 2,000 cells in G1 phase of the same cell lines. For the 2,000 cells, 17 amplification cycles were used.

**Figure 6:**
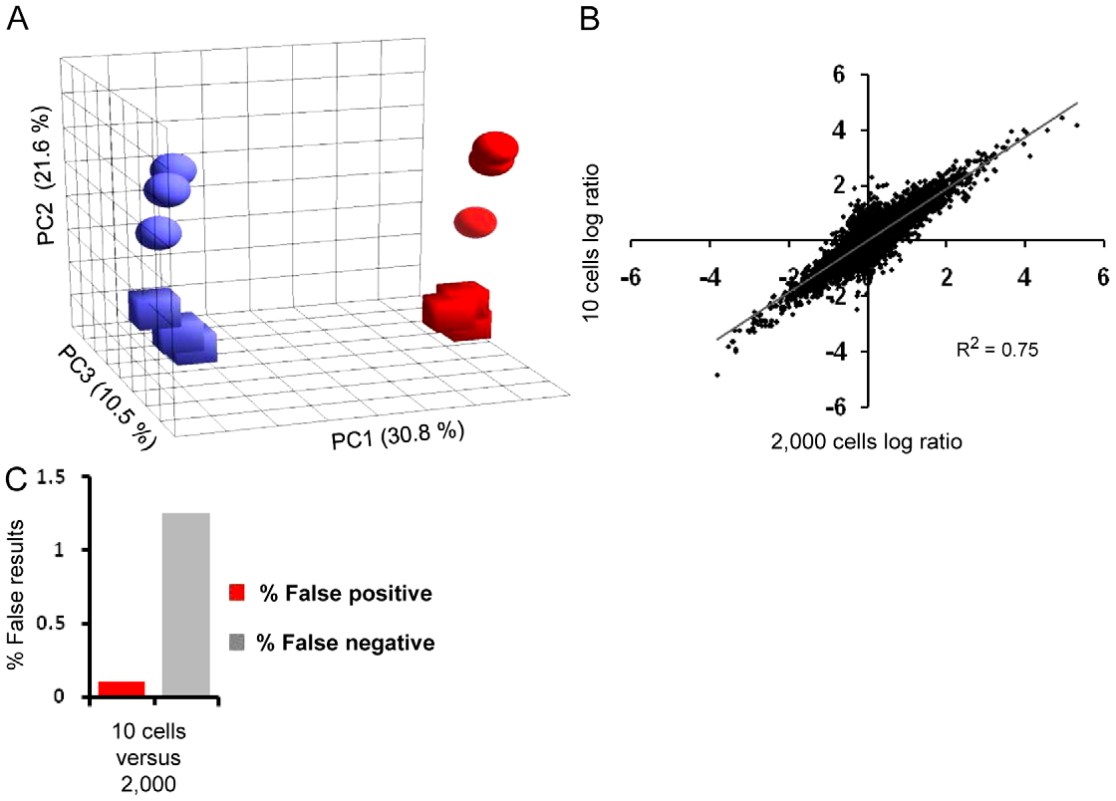
Pico Profiling from 10 cells. *A*, PCA of expression profiles of SW480 cells (red) and SW620 cells (blue) processed in triplicate starting from 2,000 cells (cubes) and 10 cells (globes); the contribution of the specific component is shown next to its axis. *B*, correlation of differential expression between SW480 and SW620 cells measured from 2,000 and 10 cells; average values of triplicates are displayed. *C*, quantification of false positive and false negative rates for expression profiling from 10 cells versus 2,000 cells.

In PCA, expression profiles from 10 cells separated the samples in a similar direction and distance as those generated from 2,000 cells (Figure 6A). Results of differential expression across all the probe sets on the Gene ST array, measured from 10 and 2,000 cells, correlated well with each other (Figure 6B). False positive and false negative rates for differential expression were in a similarly low range as for measurements from 100 pg RNA (Figure 6C). The 100 pg RNA used in the validation study of MAQC samples represents the average amount of RNA that we purified from 10 cells from various cell lines, including those addressed here for expression profiling (Table S2).

In summary, Pico Profiling fulfills the four criteria for a reliable expression profiling method described in the introduction: 1) It provides results of differential expression from diluted RNAs comparable to standard protocols (Figures 2B and 3C). 2) The measurements from diluted RNAs correlate comparably well to qPCR as the expression profiles from standard protocols do (Figure 2C and 3D). 3) The variability of technical replicates is so small that differential expression measured between samples represent biological differences (see Table 1, % outliers and Figure 3B). 4) Average measurements from RNAs purified from small cell populations provide results similar to those of big populations (Figure 6).

All data is deposited in Gene Expression Omnibus under accession code GSE20595.

## Discussion

Here we describe Pico Profiling, a method how expression profiling can accurately be generated from as few as 10 cells, and these profiles provide as much information as standard techniques, which use thousands to millions of cells. Pico Profiling also generates results of differential expression, which are highly comparable to results from standard profiling methods or qPCR, even if performed in other laboratories. We used the same amplification chemistry on other types of arrays, which harbor probes of the other orientation relative to the transcripts under interrogation (Affymetrix Mouse 430 2.0 arrays), and on this microarray platform we also obtained results that are highly comparable to those from standard processing (data not shown). We conclude that WTA-based expression profiling is suitable for analysis on a variety of microarray platforms.

While analyzing the performance of the proposed profiling method, we focused on measurements of differential expression between distinct cell types instead of absolute measurements of one cell type. This decision was based on the observation that expression profiling works best for relative differences between samples [3], [28]. When we compared absolute as well as differential expression measured from large and small amounts of starting material, we repeatedly detected slightly higher variability in data from technical replicates from smaller amounts. Once samples are extremely diluted, stochastic events may lead to variations in the number of molecules of a certain transcript in aliquots of the same RNA preparation. If this were the case, weakly expressed transcripts should be affected more greatly than those expressed at high copy numbers. The hypothesis that stochastic events cause greater variability of measurements from low amounts of starting material is supported by the observation that expression estimates of transcripts that show strong differences between replicates on average have only half the intensity of truly differentially expressed genes.

In addition, we applied our profiling method to individual cells. Strong variation in gene expression between individual cells was detected (data not shown). With our current knowledge, we cannot distinguish whether the variation between individual cells is due to unequal efficiency of amplification between samples or whether it truly represents biological differences between cells. Since the specific cell is destroyed during analysis, confirmation of expression cannot be performed by an alternative method. Also, given that we observed comparable amounts of cDNA after amplification from zero and one cell, we have opted not to pursue the analysis of individual cells.

The method proposed has several limitations. Given that microarrays contain probes only for known genes, the discovery of unknown transcripts is not possible. Alternative splicing can be analyzed only to a limited extent and only in the case of microarrays with probes for individual exons. Due to the nature of amplification method, the strand of genomic DNA from which the transcript has been generated cannot be identified. These limitations, except for the orientation of the transcript, could be overcome using massive parallel sequencing to analyze the amplification product. Unfortunately, the current WTA method generates primer sequences on both ends of the amplified cDNA fragments and these sequences would be read first when analyzed by current massive parallel sequencing methods. This would be a particular problem for the short read sequences generated by most of the technologies currently available. Also, due to using random primers for cDNA synthesis, a large fraction of sequenced molecules would represent ribosomal RNAs, which are generally not of great interest in expression profiling.

At present, the only method available to analyze transcription profiles of very few cells by sequencing [18] does not fulfill two out of four criteria for an accurate profiling method suggested in the introduction of this manuscript at all (1. diluted samples should provide comparable results as the concentrated samples do when standard methods are used; 2. diluted samples should provide results by profiling comparable to qPCR; 3. technical replicates from the same RNA preparation should provide small differences in results; 4. small cell populations should provide results similar to bigger populations). Tang *et al*. do not describe experiments, which evaluate criteria 1 and 4. In their comparison of sequencing vs. qPCR expression data, (criterion 2) Tang *et al*. excluded approximately 70 percent of the measurements from the comparison due to a criterion based on qPCR (only the top 100 most abundant genes based on the Ct values obtained in qPCR out of 378 measured genes were compared to sequencing results in Supplementary Figure 6 of the original publication). This means that for the remaining transcript measurements, which were not validated by qPCR, no criterion is available on reliable versus non-reliable results. In a profiling experiment aimed to discover biological differences, qPCR data would not be available for the majority of measurements. When we re-analyzed the Tang data without the filtering criterion based on qPCR, sequencing generated high rates of false positive and false negative measurements (Figure 4C). Technical reproducibility (criterion 3) was evaluated solely on different biological samples, a design which does not allow separating technical variation from biological variation. Therefore, we conclude that currently no well-evaluated expression profiling method for characterization of very small cell populations by sequencing is available.

The possibility to accurately characterize the expression profiles of extremely small populations of cells by Pico Profiling will increase our understanding of many fields of biology, including stem cells, early embryonic development, homing of metastatic cancer cells, and other areas of biology which attempt to characterize specialized cells, which are only available in small numbers.

## Materials and Methods

### Whole Transcriptome Amplification (WTA) and Pico Profiling

For RNA amounts of 25 or 50 ng, as used initially and in the 2,000 cell analysis, library preparation and amplification for 17 cycles were performed following the distributor's (Sigma-Aldrich) recommendations. For amplification of smaller amounts of RNA, SYBR Green and ROX (both Sigma-Aldrich) were added to the amplification reaction, which was performed in a 7900 HT Real-time instrument (Life Technologies) to monitor amplification yield. Once the SYBR Green signal reached a plateau, the reaction was stopped. Amplified cDNA was purified and quantified on a Nanodrop ND-1000 spectrophotometer (Thermo-Fischer). 10 µg cDNA was subsequently fragmented by DNAseI and biotinylated by terminal transferase obtained from GeneChip Mapping 10Kv2 Assay Kit (Affymetrix). Hybridization, washing, staining and scanning of Affymetrix Human Gene ST 1.0 arrays were performed following the manufacturer's recommendations (http://media.affymetrix.com/support/downloads/manuals/wt_dble_strand_target_assay_manual.pdf). A detailed protocol is available in Supplementary Methods.

### RNA isolation using magnetic beads

To obtain more homogeneous cell populations, viable cells were stained by HOECHST 33342 (Sigma-Aldrich) for their DNA content and enriched for a fraction in G1 of the cell cycle using a FACS Aria SORP cell sorter (Becton Dickinson). Cells were sorted into lysis buffer. Directly after cell sorting, the plate containing lysis buffer and lysed cells was incubated for 15 minutes at 65°C. Subsequently, RNA was purified using RNA Clean XP bead suspension (Agencourt Bioscience). Genomic DNA is bound by the beads but released in only a small percentage as long as it is not sheared to small fragments (data not shown). Therefore, every effort was made to minimize shearing caused by pipetting. RNA was eluted in 22 µl water and 19.1 µl of this solution was used for WTA. For the RNA preparations from thousands of cells, RNA was quantified using the Quant-iT RNA Assay kit (Life Technologies). A detailed protocol for RNA isolation is available in File S1.

### Data analysis of Gene ST 1.0 arrays

Scanned images (DAT files) were transformed into intensities (CEL files) by GCOS (Affymetrix). These arrays contain probe sets intended to measure ribosomal RNAs, miRNAs, tRNAs and other small RNAs, as well as positive and negative control probe sets. Since our amplification and labeling method is not intended for use with these types of RNAs, these probe sets were excluded from normalization. Afterwards, quantile scaling and WPP algorithm were used to normalize and calculate expression estimates [29]. The software for filtering and WPP normalization can be downloaded from www.dnaarrays.org. PCA was performed by means of the Genomics Suite (Partek).

### Reanalysis of published data sets and comparison to WTA-based expression profiles

Rawdata (CEL files) of Shi et al. [3] (Affymetrix Laboratory 1 of the MAQC study), Pradervand et al. [25] and dChip expression estimates of Kurimoto et al. [17] were downloaded from Gene Expression Omnibus (GSE5350, samples GSM122774-76, GSM122779-81, GSE9819 and GSE4308 respectively). Replicates one, two and three were used when more than three replicates were available. The first three replicates were also used from the CEL files of Jensen and Watt [20], when they measured expression profiles from 50 pg RNA and compared these with results obtained from the identical samples, which were processed by Wilson et al. [26], using the standard protocol starting from 5 µg RNA. For these data sets, RMA normalization was performed using the Genomics Suite (Partek). Since Wilson's data set used an array (U133 Plus 2.0) that contains more probe sets than the one used for Jensen's data set (U133A), only the probe sets also present on the U133A array were used for further analysis. qPCR data of Laboratory 1 of the MAQC study [3] were downloaded from Gene Expression Omnibus (GSE5350, samples GSM129638-44). Data were power-transformed on the basis of 2 to generate ΔCt values. For comparison with microarray data, values of quadruplicates were averaged and the ΔΔCt value was calculated by subtracting the ΔCt of URR from brain RNA.

For correlation analysis, results of microarray and qPCR replicates were averaged. When multiple probe sets for the same transcript were available on the microarray or multiple measurements of the identical transcript were available in a PCR data set, the probe set or PCR assay showing the highest absolute value of differential expression was used.

From Tang's [18] data set, data of Supplementary Table 3 of the original publication was used, specifically the normalized transcript counts and Ct values of Refseq transcripts. For RT-PCR data, duplicates of wild-type oocyte 1 and dicer −/−1 were each averaged and dicer−/− values were subsequently subtracted from wild-type values. For sequencing data, normalized counts of transcripts from wild-type 1 and dicer −/−1 were log2 transformed, after counts of zero had been replaced by one and wild-type 1 was subtracted from dicer −/−1. For comparison with Real-time PCR results, only transcripts with a minimal count of five sequences were used. This cutoff was proposed in the original publication.

### Partitioning of expression profiling results

To evaluate the performance of different expression profiling methods, Two replicate groups of log2-transformed expression measurements are classified as “positive”, “negative”, “outlier” or “ambiguous” depending on the distribution of between-group differences:

- “positive” if all differences have the same sign and have magnitude greater than 1;

- “negative” if all differences have magnitude less than 1;

- “outlier” if not all differences have the same sign and some differences have magnitude greater than 2;

- “ambiguous” if none of the above.

Measurement results are categorized according to test and reference classifications:

- “TruePositive” if both test and reference are “positive” with the same sign;

- “FalseNegative” if test is “negative” and reference is “positive”;

- “PositiveOutlier” if reference is “positive” and test is either “outlier” or “positive” with opposite sign;

- “TrueNegative” if both test and reference are “negative”;

- “FalsePositive” if test is “positive” and reference is “negative”;

- “NegativeOutlier” if test is “outlier” and reference is “negative”;

- “Undecided” if none of the above.

Counts of the first three categories are normalized by their sum (total reference “positive”) and, similarly, counts of the next three categories are normalized by their sum (total reference “negative”). “Undecided” results are not counted.

## Supporting Information

Figure S1 Size distribution of amplified cDNAs from different amounts of starting material. Typical electropherograms of WTA amplified cDNAs from 0 pg RNA (A), 10 pg (B), 100 pg (C) and 1000 pg RNA (D).
(0.32 MB TIF)

Figure S2 Probe signal intensities of microarrays hybridized with cDNA generated from different amounts of RNA. 10μg cDNA was generated by WTA amplification from the indicated amounts of RNA and hybridized to Gene ST arrays. Whiskers indicate range, boxes the 25th and 75th percentile, and horizontal lines within boxes indicate the median.
(0.08 MB TIF)

Figure S3 Influence of expression levels on the correlation of microarray versus qPCR measurements of differential expression. Transcripts were divided into high-copy-number and low-copy numbers according to the Ct values from qPCR measurements. Correlation of qPCR measurements versus microarray measurements for high abundance transcripts (A and C) and low abundance transcripts (B and D) measured from 25 ng RNA (A and B) and 100 pg respectively (C and D).
(0.23 MB TIF)

Figure S4 Integrity of RNA after magnetic bead purification. Typical electropherograms of RNA isolated from (A) SW480 and (B) SW620 cells.
(0.13 MB TIF)

Table S1 (0.03 MB DOC)

Table S2 (0.03 MB DOC)

File S1 (0.14 MB PDF)
