# Supplementary figures and images for: Accurate Expression Profiling of Very Small Cell Populations
Source: PLoS One. 2010 Dec 28;5(12):e14418. doi: 10.1371/journal.pone.0014418 (PMC3010985; doi:10.1371/journal.pone.0014418)

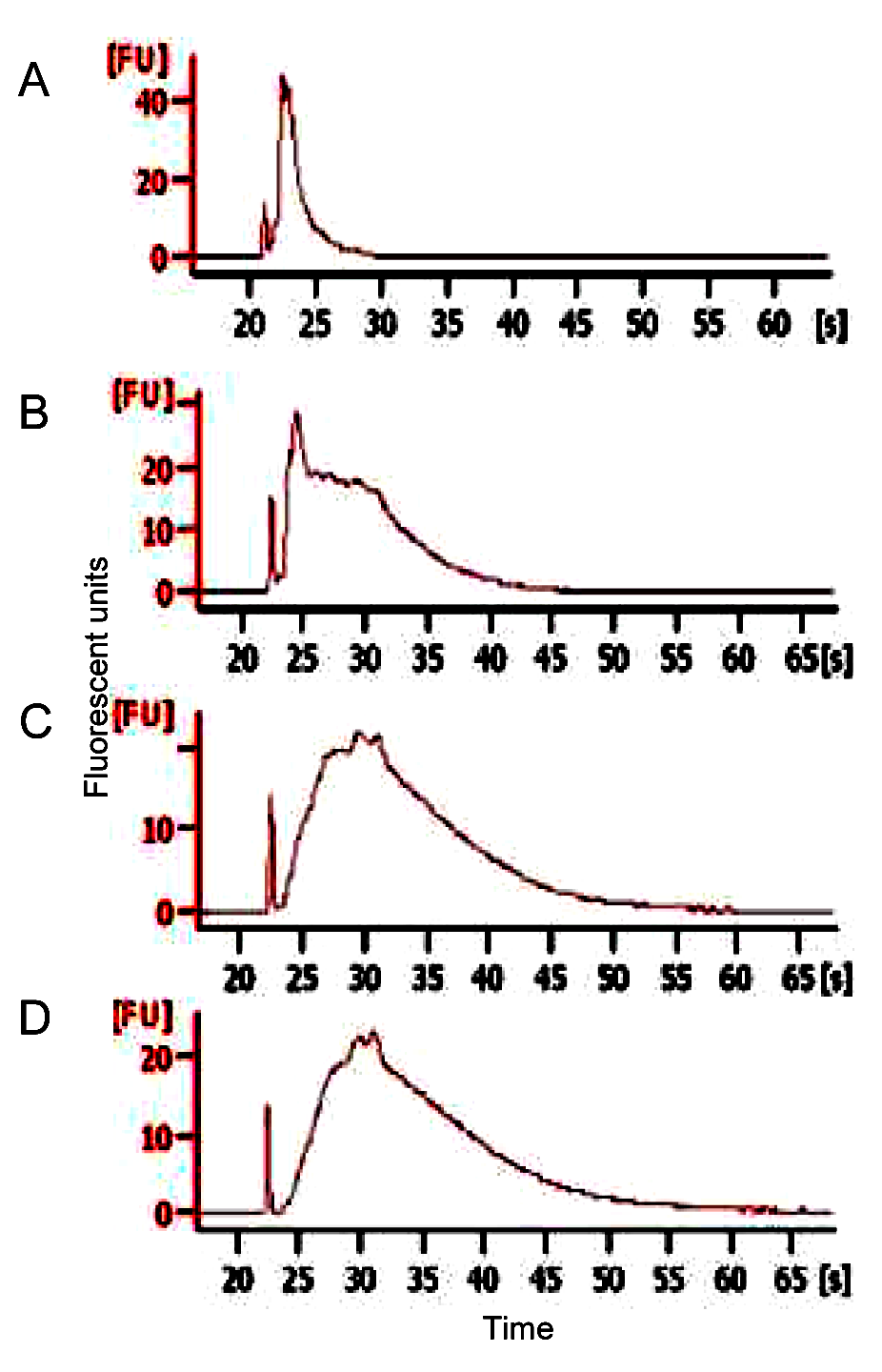

Supplement: Figure S1 — Size distribution of amplified cDNAs from different amounts of starting material. Typical electropherograms of WTA amplified cDNAs from 0 pg RNA (A), 10 pg (B), 100 pg (C) and 1000 pg RNA (D). (0.32 MB TIF) [file pone.0014418.s001.tif]

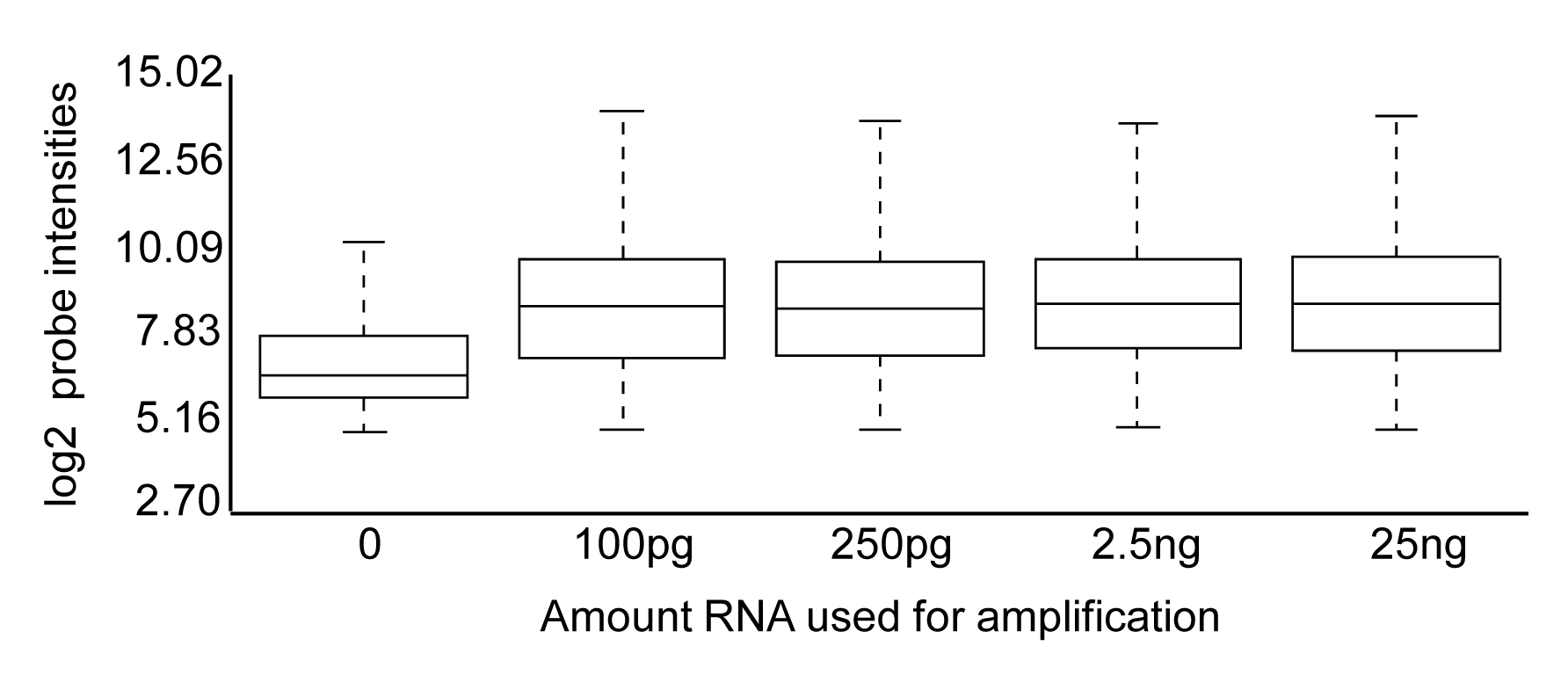

Supplement: Figure S2 — Probe signal intensities of microarrays hybridized with cDNA generated from different amounts of RNA. 10μg cDNA was generated by WTA amplification from the indicated amounts of RNA and hybridized to Gene ST arrays. Whiskers indicate range, boxes the 25th and 75th percentile, and horizontal lines within boxes indicate the median. (0.08 MB TIF) [file pone.0014418.s002.tif]

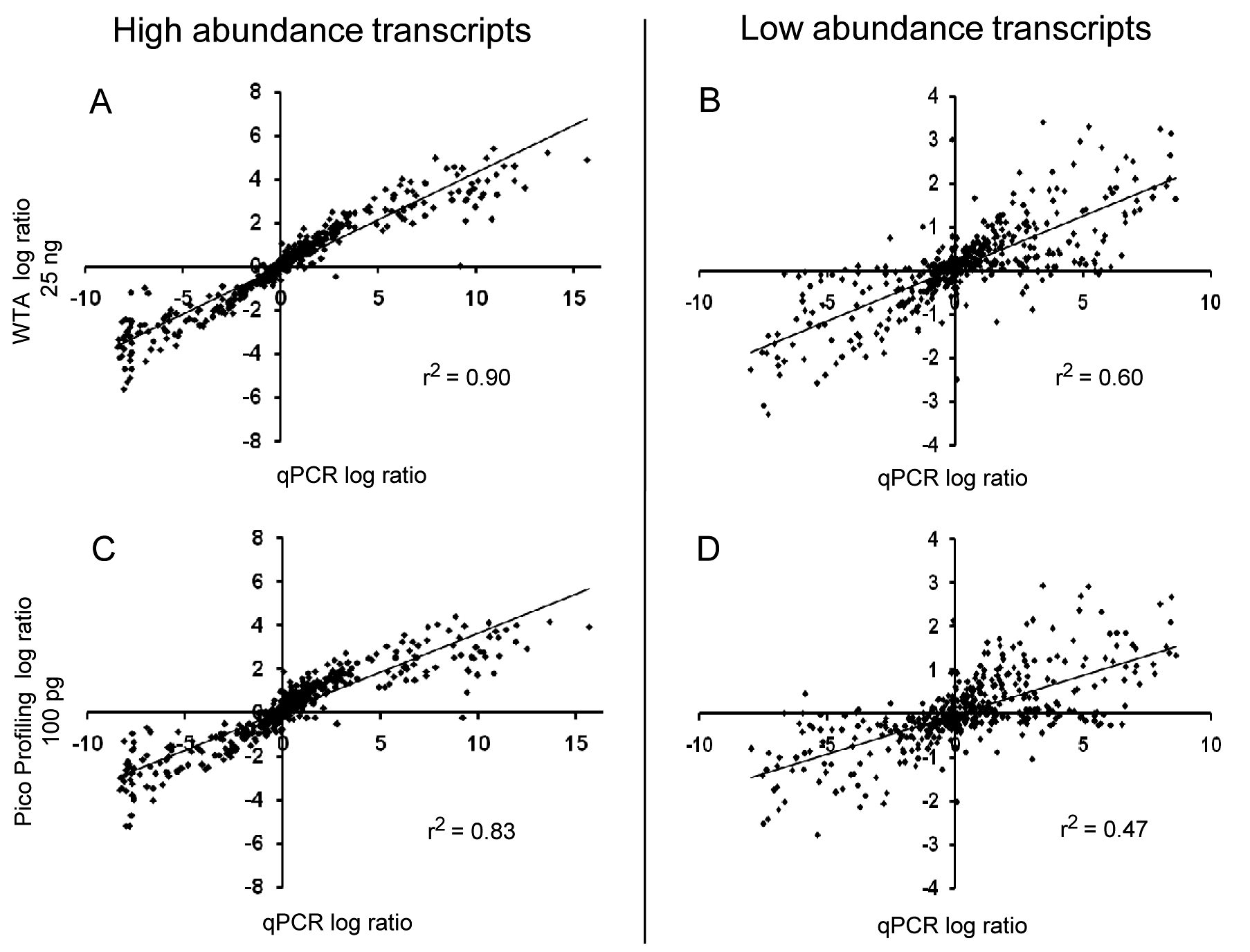

Supplement: Figure S3 — Influence of expression levels on the correlation of microarray versus qPCR measurements of differential expression. Transcripts were divided into high-copy-number and low-copy numbers according to the Ct values from qPCR measurements. Correlation of qPCR measurements versus microarray measurements for high abundance transcripts (A and C) and low abundance transcripts (B and D) measured from 25 ng RNA (A and B) and 100 pg respectively (C and D). (0.23 MB TIF) [file pone.0014418.s003.tif]

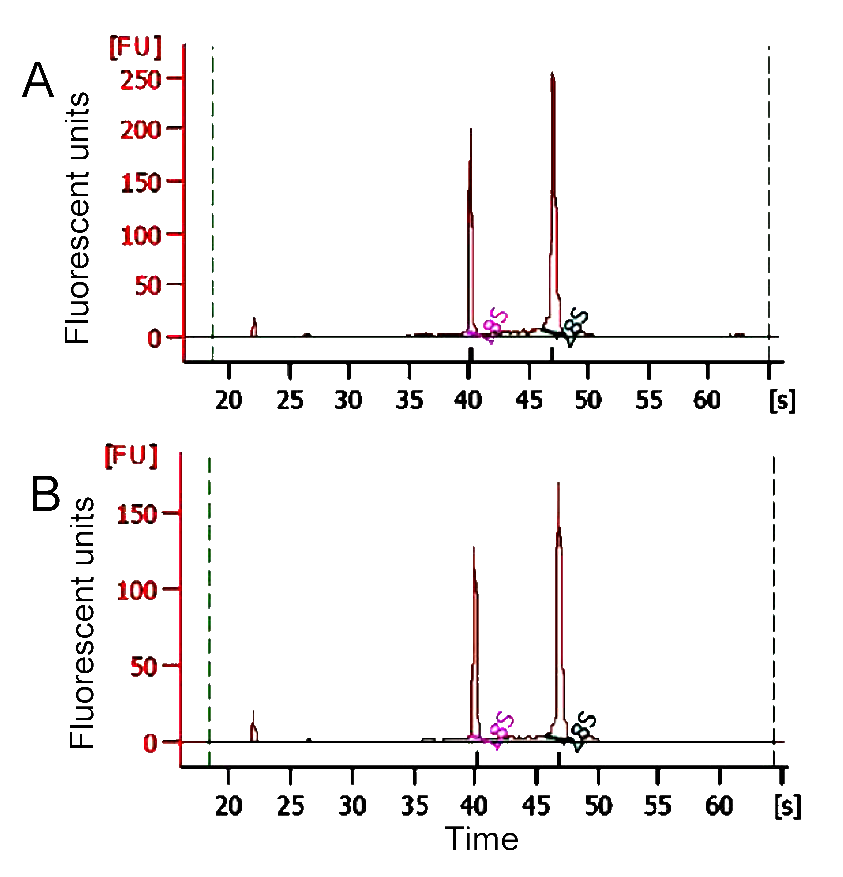

Supplement: Figure S4 — Integrity of RNA after magnetic bead purification. Typical electropherograms of RNA isolated from (A) SW480 and (B) SW620 cells. (0.13 MB TIF) [file pone.0014418.s004.tif]
