# Supplementary material for: Accurate Expression Profiling of Very Small Cell Populations
Source: PLoS One. 2010 Dec 28;5(12):e14418. doi: 10.1371/journal.pone.0014418 (PMC3010985; doi:10.1371/journal.pone.0014418)
Supplement: Table S1 — (0.03 MB DOC) [file pone.0014418.s005.doc]

**Supplementary Table 2. RNA yield per cell after magnetic bead purification from 1,000 to 5,000 cells of various cell lines**

| Cell type | pg RNA/cell |
| --- | --- |
| SW620 | 8.7 |
| SW480 | 16.3 |
| Bruce4 | 15.2 |
| KM12C | 13.4 |
| NSO | 9.7 |
