# Supplementary material for: Accurate Expression Profiling of Very Small Cell Populations
Source: PLoS One. 2010 Dec 28;5(12):e14418. doi: 10.1371/journal.pone.0014418 (PMC3010985; doi:10.1371/journal.pone.0014418)
Supplement: File S1 — (0.14 MB PDF) [file pone.0014418.s007.pdf]

Magnetic Bead Purification of RNA

Note: Once RNA is purified, library preparation and amplification has to be done immediately. Diluted RNA/cDNA solutions should not be frozen. The entire process takes 8-9 hours and a maximum of 16 amplification reactions for Pico Profiling can be performed per Real-time PCR machine.

Lysis buffer: (20 mM DTT, 10 mM Tris.HCl ph 7.4, 0.5% SDS, 0.5µg/µl proteinase K) Proteinase K digests itself; therefore, keep lysis buffer as shortly as possible at room temperature!

1. Add 45 µl lysis buffer per well into a V-bottomed 96-well polypropylene plate (Greiner Bio-One) for each aliquot of cells to be purified; add some aliquots to some more wells in case there is a problem with the cell sorting. **Note**: Do not use a PCR plate here; the small diameter of PCR wells does not allow efficient mixing by shaking the plate!

2. Sort 10 to 2,000 cells into each well; the total volume of sorted cells may not exceed 5 µl.

3. Directly after cell sorting, seal the plate containing lysis buffer and lysed cells and incubate for 15 minutes at 65°C in a water bath. One lysis buffer aliquot must not contain any cells. This aliquot serves as a negative control during amplification.

4. Bring RNA Clean XP bead suspension (Agencourt Bioscience) to room temperature. Add 90 µl of bead suspension to each aliquot of lysis buffer (with and without lysed cells). Do not pipette up and down. **Note**: Genomic DNA is bound too by the beads but it is released very poorly as long as it is not sheared into small fragments.

5. Seal the plate and mix gently by shaking the plate. This can be done by turning on a vortex and touching it with the side of the plate. Check that beads form a homogenous suspension and leave 5 minutes at RT.

6. Place plate on top of a SPIR magnetic stand (Agencourt Bioscience) and fix with an adhesive tape so that plate and magnetic stand are tightly connected. Leave the plate on the magnetic stand for 5 minutes.

7. **Critical step:** First removal of supernatant: At this time, beads do not stick well to the magnet. Remove supernatant with 200 µl pipette, but leave approximately 10 µl liquid on top of the beads. For the remaining liquid use a 10 µl pipette with a fine tip; remove as much liquid as possible without removal of beads.

8. Add 180 µl 75% EtOH (mixed freshly) to the beads without pipetting up and down. The plate stays on the magnetic stand. Remove liquid by pipetting; by now, the beads stick well to the magnet. Repeat the EtOH wash and remove remaining liquid completely. Dry 5 minutes at RT.

9. Remove the plate from the magnet and add 22 µl water to each bead well. Do so by placing the water over the beads and shake the plate gently. Leave 5 minutes at RT.

10. Put plate on magnet and leave it there for 5 minutes. Use 19.1 µl of this RNA preparation for Pico Profiling. If RNA was purified from > 1,000 cells, the remaining liquid can be used for Qubit quantification (Quant-iT RNA Assay kit (Life Technologies)).

11. Proceed immediately with library synthesis and amplification

Pico Profiling

1 Library Synthesis and Amplification

TransPlex® Complete Whole Transcriptome Amplification Kit (WTA2-50RXN, Sigma-Aldrich) is used for this protocol.

A. Library Synthesis Reaction (in regular PCR machine)

Thaw the Library Synthesis Buffer, Library Synthesis Solution at room temperature. Mix by vortexing for 2 seconds, spin in a microcentrifuge for 2 seconds and place on ice. Dissolve any precipitate in these solutions by briefly heating at 37 ℃, with thorough mixing.

1. Pre-heat the thermocycler by starting the following program (“anneal”) and pause it once it reaches 70ºC.
   - 70ºC for 5 minutes
   - 18ºC hold
2. Place 0.2 ml PCR tube strip in a rack on ice. For each reaction, pipette 2.5µl Library Synthesis Solution into each tube.
3. Add 19.1µl of bead purified RNA. Immediately place the tubes in the pre-heated thermal cycler (70ºC). Run the “anneal” program. Let the reaction cool down to 18ºC and add immediately the Library Mix (see below). **Critical point!**
4. Prepare Library Mix as outlined below.

Library Synthesis Buffer	2.5
Water	3.9
Library Synthesis Enzyme	2
**Total**	8.4

5. **Immediately** add 8.4 µl of Second Library Mix to the cooled annealed RNA. Run the following program (“library”):
   - 18ºC for 10 minutes
   - 25ºC for 10 minutes
   - 37ºC for 30 minutes
   - 42ºC for 10 minutes
   - 70ºC for 20 minutes
   - 4ºC hold

B. Library Amplification (in Real-time PCR machine)

1. While the library synthesis is running thaw Amplification Mix Buffer and WTA dNTP Mix at room temperature, mix by vortexing for 2 seconds and then spin in a microcentrifuge for 2 seconds. Place on ice.
2. Prepare fresh reference dye (ROX) and SYBR® Green 10x dilutions. Reference dye (ROX) (R4526-.3ML, Sigma-Aldrich) is 100x concentrated and SYBR® Green (S9430-.5ML, Sigma-Aldrich) is 10,000x concentrated.
3. Spin the strip from step A8 and transfer each entire Library Synthesis reaction (30 µl) into a 1.5 ml tube on ice.
4. Prepare the Amplification mix as outlined below.

Water	293.5
Amplification Mix Buffer	37.5
10 mM dNTP mix	7.5
Reference dye (ROX) 10x	3.75
SYBR Green 10 x	3.75
Amplification Enzyme	3.75
**Total**	349.8

5. Add 349.8 µl mix to each sample, mix well by pipetting up and down. Split each reaction into five 75 µl reactions.
6. Load a 96-well plate suitable for a real-time machine as follows:

	**1**	**2**	**3**	**4**	**5**	**6**	**7**	**8**	**9**	**10**	**11**	**12**
**A**	1	1	1	1	1		9	9	9	9	9	
**B**	2	2	2	2	2		10	10	10	10	10	
**C**	3	3	3	3	3		11	11	11	11	11	
**D**	4	4	4	4	4		12	12	12	12	12	
**E**	5	5	5	5	5		13	13	13	13	13	
**F**	6	6	6	6	6		14	14	14	14	14	
**G**	7	7	7	7	7		15	15	15	15	15	
**H**	8	8	8	8	8		16	16	16	16	16	

7. Seal the plate with an optical film and spin it for 1 min at 1,000 rpm.
8. Run the "amplify" program (94ºC for 2 minutes, 40 cycles (94ºC for 30 sec, 70º for 5 min, data collection at 70º)) in the real-time PCR machine and stop the reaction once the SYBR-Green signals have reached the plateau phase.

C. Cleanup of Double-Stranded cDNA

ZYMO DNA clean & concentrator-25 (ref. D4006, Zymo Research) is used in this step.

1. Add 750 µl of DNA Binding Buffer to a new 1.5 ml tube.
2. Transfer the complete volume of every amplification reaction by recovering all five 75 µl from each sample into the previously filled 1.5 ml tube.
3. Mix by vortexing for 3 seconds o by pipetting. Spin.
4. Apply 600 µl of the sample to the Zymo Spin II Column sitting in a 2ml Collection Tube (supplied), and centrifuge for 10 seconds at ≥ 10,000 X *g*. Discard flow-through.
5. Reload the spin column with the remaining mixture and centrifuge as above. Discard flow-through.
6. Wash sample by adding 200 µl of room temperature 80% ethanol. **Do not use** the wash buffer provided with Zymo columns. **NOTE:** *Use fresh 80% ethanol. Lower percent ethanol mixes will reduce recovery.*
7. Centrifuge column in the collection tube for 10 seconds at 10.000 x g in a microcentrifuge. Discard flow-through.
8. Wash again with 200 µl of room temperature 80% ethanol. Centrifuge column in the collection tube for 10 seconds at 10.000 x g in a microcentrifuge. Discard flow-through and collection tube.
9. Blot the column tip onto a filter paper to remove any residual wash buffer from the tip of the column. **NOTE:** *Blot column tip prior to transferring it to a new tube to prevent any ethanol transfer to the eluted sample*.
10. Transfer spin column into a new 1.5 ml tube, and pipet 30 µl of room temperature nuclease-free water included in the WTA2 kit from Sigma directly to the center of the spin column membrane. **Do not user cold water**!
11. Incubate for 1 minute at RT and spin column and microcentrifuge tube for 30 seconds at 10.000 x g in a microcentrifuge. There should be approximately 30 µl of purified dsDNA. Mix sample by vortexing, the spin briefly.

TIP: Label the collection tubes with the sample name. During centrifugation some column caps may break, resulting in loss of sample information.

TIP: Place columns into the centrifuge using every second bucket. Position caps oriented in the opposite direction to the rotation.

12. Proceed to measuring dsDNA yield and purity using the Nanodrop spectrophotometer. There must be more than 10 µg per sample (usually 13-15 µg). The negative control should give less than half of the yield of 10 cells.
13. **STOP point**: Keep samples at -20ºC or proceed to next step (Fragmentation and Labeling).

D. cDNA Fragmentation

1. Calculation of cDNA Input Volume

The default cDNA amount to be fragmented is 10 µg, but the overall amplification efficiency must be considered. After cDNA cleanup two scenarios are possible:

a) cDNA concentration more than 445 ng/µl. Take 22.5 µl for fragmentation.
b) cDNA concentration less than 445 ng/µl. Take 10 µg and bring it to 22.5 µl by speed-vac.

2. Fragmentation Protocol

Box 3 of the GeneChip Mapping 10K Xba Assay Kit (ref. 900441, Affymetrix) is used for this step.

Note added in proof: Affymetrix plans to discontinue the 10K Xba Assay Kit as a list product by 2011. Custom orders will be produced for > 50 kits. Alternatively, individual components can be purchased separately from Affymetrix:

14367 100 UN ,DNase I, Solution, 100 units

72033 500 UN, Terminal Deoxynucleotidyl Transferase (rTdT), Recombinant, 500 units, comes with buffer

79015 250 NM, Biotin-11-dXTP Analog (DNA Labeling Reagent, DLR), 250 nmol (10 mM)

UPDATED INFORMATION ON CONDITIONS OF USE OF THESE INDIVIDUAL COMPONENTS WILL BE AVAILABLE SOON ON THE DOWNLOADS PAGE OF www.dnaarrays.org.

1. Pre-heat thermal cycler to 37°C.
2. Thaw the 10X Fragmentation Buffer at room temperature and mix by vortexing for 2 seconds and then spin in a microcentrifuge for 2 seconds. Then, place on ice.
3. Mix the Fragmentation Reagent by inverting the tube 3 times. Manually spin down the tube. Keep in the cooler at -20ºC.

4. Dilute the stock of Fragmentation Reagent to 0.048 U/µL using Fragmentation Buffer and Molecular Biology Water **on ice** and vortex at medium speed for 2 seconds. Examine the label of the GeneChip Fragmentation Reagent tube for U/µL definition, and calculate dilution. Three examples of dilution are listed below for different concentrations of Fragmentation Reagent.

NOTE: *As the concentration of stock Fragmentation Reagent (U/µL) may vary from lot to lot, it is essential to check the concentration before conducting the dilution. Do calculations prior to diluting Fragmentation Reagent.*

NOTE: *The Fragmentation Reagent is viscous so it is important that it is mixed well.*

	**2 U/µl**	**2.5 U/µl**	**3 U/µl**
Fragmentation Reagent	3	2.5	2
10x Fragmentation Buffer	12.5	12.5	12.5
Water	109.5	110	110.5
**Total**	125	125	125

5. Place 0.2 ml PCR tube(s) in a rack on ice. For each reaction, pipette 22.5 µl (10 µg) of the purified DNA into a PCR tube.
6. Make Fragmentation Master Mix **on ice** as outlined below:

DNA (10 µg)	22.5
10x Fragmentation Buffer	2.5
Diluted Fragment Reagent	2.5
**Total**	27.5

7. Add 5 µl of the Fragmentation Master Mix to each sample. Vortex at medium speed for 2 seconds. Place back on ice.
8. Immediately place the tubes in pre-heated thermal cycler (37°C). Run the following program („snpfragment“):
   a. 37ºC for 30 minutes
   b. 95ºC for 15 minutes
   c. 4ºC Hold
9. Spin the tube(s) briefly after fragmentation reaction.

10. Proceed immediately to Labeling step.

E. Labeling Protocol

1. Thaw the 5X TdT Buffer and the GeneChip DNA Labeling Reagent at room temperature, mix by vortexing for 2 seconds and then spin for 2 seconds. Then, place on ice.
2. Make Labeling Master Mix **on ice** as outlined below:

5x TdT Buffer	7
DNA Labeling Reagent	1
TdT (30 U/µl)	1.7
**Total**	9.7

3. Add 9.7 µl of the Labeling Master Mix to each sample. Vortex at medium speed for 2 seconds. Place back on ice.
4. Immediately place the tubes in pre-heated thermal cycler (37°C). Run the following program („snplabeling"):
   a. 37ºC for 2 hours
   b. 95ºC for 15 minutes
   c. 4ºC Hold

NOTE: *Make sure the reaction tubes are securely sealed prior to running this program in order to minimize solution loss due to evaporation at denaturation step (95°C).*

5. Spin the tube(s) briefly after fragmentation reaction. Samples can be stored at -20ºC if not, immediately proceeding to next step.

F. Target Hybridization

Use GeneChip Hybridization, Wash and Stain Kit (ref. 900720) from Affymetrix in the following steps.

1. Pre-warm the oven at 45ºC with the rotor at approximately 30 rpm. Allow the arrays to equilibrate at RT.

2. Obtain the 3' Amplification Hybridization Control kit (Oligo B2, 20x Hyb control, ref 900454, Affymetrix) stored at -20ºC, thaw at room temperature and mix by vortexing for 2 seconds and spin down. Then, place on ice.
3. Heat the 20X GeneChip Eukaryotic Hybridization Controls to 65℃ for 5 minutes to completely resuspend the cRNA. Place them on ice until their use.
4. Make Hybridization Cocktail Master Mix as outlined below:

Control Oligo B2	2
20x Hyb Controls	6
2x Hybridization solution	60
DMSO	8.4
Water	7
**Total**	83.4

5. Add 83.4 µl of the Hybridization Cocktail Master Mix to each sample. Label the tubes as "HYB" after the sample ID. *This Hybridization Mix can be stored at -20ºC before proceeding to the next step*.
6. Denature the Hybridization Mix at 99ºC for 2 minutes in a heat block to denature.
7. Transfer to 45℃ for 5 minutes.
8. Spin briefly and vortex at low rpm.
9. Vent the array chamber by inserting a clean, unused pipette tip into one of the septa (see Figure below for location of the probe array septa).

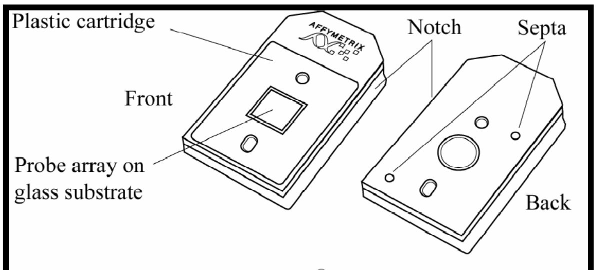

Figure 1 GeneChip® Probe Array

10. Insert the pipette tip of a micropipettor into the remaining septum and fill the array with the appropriate volume of the clarified hybridization cocktail, <u>avoiding any insoluble matter at the bottom of the tube</u>.
11. Place probe array into the hybridization oven, set to 45℃. To avoid stress to the motor, load probe arrays in a balanced configuration around the axis. Rotate at 60 rpm.
12. Hybridize over night (16-18h).

G. Washing and Staining

Use FS450_0007 program in the fluidics station 450 from Affymetrix. Use the regular buffers from the GeneChip® Hybridization, Wash and Stain Kit (Wash A and B).
